# Region-Specific Variation in the Electrophysiological Responses of *Spodoptera frugiperda* (Lepidoptera: Noctuidae) to Synthetic Sex Pheromone Compounds

**DOI:** 10.1007/s10886-024-01479-w

**Published:** 2024-02-29

**Authors:** Mobolade D. Akinbuluma, Renée A. H. van Schaijk, Peter Roessingh, Astrid T. Groot

**Affiliations:** 1 Department of Evolutionary and Population Biology, University of Amsterdam, Amsterdam, The Netherlands; 2 Department of Crop Protection and Environmental Biology, University of Ibadan, Ibadan, Nigeria

**Keywords:** Fall armyworm, Electroantennogram, Cytochrome oxidase I gene, Mitotype

## Abstract

The fall armyworm, *Spodoptera frugiperda* (J.E. Smith), is a global pest that feeds on  > 350 plant species and causes major yield loses. Variation in the responses of *S. frugiperda* males to female sex pheromone compounds affects the detection, monitoring and management of the pest. We determined geographic variation in the responses of *S. frugiperda* males to four different doses of synthetic sex pheromone compounds using a gas chromatography-electroantennogram detector (GC-EAD). Furthermore, we disentangled regional populations into C- and R- mitotypes via molecular analysis of the cytochrome oxidase I gene, and measured their responses to the compounds. When comparing responses of males from Florida, Benin, Nigeria and Kenya, we found some regional differences in the responses of *S. frugiperda* males to the major compound, Z9-14:OAc and minor component Z9-12:OAc. However, we found no differences in male responses between the different African countries. All males showed significantly higher antennal responses to Z7-12:OAc than to E7-12:OAc. When comparing the mitotypes, we found that Florida R-type males showed higher responses to Z9-14:OAc, Z7-12:OAc and Z9-12:OAc than Benin R-type males, while C-type males from both regions responded equally to Z7-12:OAc. In addition, Florida R-type males showed higher responses to E7-12:OAc than Florida C-type males. Our study thus shows some differential physiological responses of *S. frugiperda* males towards the known sex pheromone compounds, including E7-12:OAc, but mostly in the different mitotypes. How these differences translate to field trap catches remains to be determined.

**Supplementary Information:**

The online version contains supplementary material available at 10.1007/s10886-024-01479-w.

## Introduction

The fall armyworm, *Spodoptera frugiperda*J. E. Smith (Lepidoptera, Noctuidae) is native to the Americas and currently feeds on a large variety of agricultural crops, belonging to 76 plant families (Montezano et al. 2018). The first report ofits invasion to other parts of the world was in 2013 in Sao Tomé (IPCC 2021), followed by Western Africa in 2016 (Goergen et al. 2016), India (Sharanabasappa et al. 2018), Asia (Bhusal and Bhattarai 2019; Sun et al. 2021) and Australia (IPPC 2021; Paudel Timilsena et al. 2022); Tay et al. 2023). Recently, it was also found on the Canary Islands in Southern Europe (IPPC 2021; Gilioli et al. 2023). Its spread is projected to continuewith climate change, especially within the Africa region (Paudel Timilsena et al. 2022). Its global spread is likely facilitated by its high migratory behavior (Westbrook et al. 2016; Baudron et al. 2019; Gilioli et al. 2023), human-assisted transport and commerce (Cock et al. 2017). In sub-Saharan Africa, one of its major hosts is maize (*Zea mays* L.), which is the most important staple food and is critical for food security of the continent (Pardey et al. 2016; Badu-Apraku and Fakorede 2017; VIB 2017). *Spodoptera frugiperda* causes up to 21 million tonnes annual reduction in maize yield (Abrahams et al. 2017), representing about 52% annual production loss (Chimweta et al. 2020). Thus, it is crucial to develop and implement evidence-based control measures for *S. frugiperda* in Africa (Prasanna et al. 2018).

The traditional approach of pesticide use has substantial environmental and human health issues as well as causing damage to non-target organisms, including natural enemies of insect pests (Desneux et al. 2007; Régis Ahissou et al. 2021). Furthermore, success of pesticides is limited in controlling *S. frugiperda* as the larvae tend to conceal themselves when feeding on the host plant by hiding in the maize whorl (Harrison et al. 2022), and resistance has been developed against many of the cheapest and most widely used pesticides in Africa (Day et al. 2017; Akeme et al. 2021). In addition, significant expenditures are incurred by both farmers and government in controlling infestation by *S. frugiperda,* thus increasing the cost of crop production. For example, in Ethiopia in the cropping season of 2017 about 300, 000 L of insecticides were used and total cost for pest management exceeded US$ 4.5 million (Kassie et al. 2020). In the same year, farmers in Zimbabwe received about 102,000 L of pesticide valued at 1.97 million US dollars to contain the spread of *S. frugiperda* (Rwomushana et al. 2018). Therefore, it is essential to develop an environmentally-friendly and more effective approach to combat the improper use of pesticides.

The application of synthetic species-specific sex pheromones is a helpful tool in integrated pest management (IPM) for monitoring and early detection of *S. frugiperda* (Prasanna et al. 2018; FAO 2019; Matova et al. 2020). However, for a successful monitoring system, it is essential that lures are attractive. In the Western Hemisphere, *S. frugiperda* is subdivided into two strains, the so-called corn strain and rice strain (Pashley et al. 1992; Lu and Adang 1996; Gouin et al. 2017), which differ by mitochondrial markers (Nagoshi 2010) as well as by nuclear differences (Gouin et al. 2017). Since strain identification is only by molecular markers and not morphological differences, it is difficult to describe the two strains with respect to their behavior in the field, culminating into several contradictory findings (Juárez et al. 2014; Nagoshi and Meagher 2022). Therefore, we refer to these strains as (C)- and (R)- strains.

Even though some differences in sexual communication has been found between the two *S. frugiperda* strains (Unbehend et al. 2013), geographic variation in response to the signals has also been found (Unbehend et al. 2014). Furthermore, although the effluvia and gland pheromone extracts from calling *S. frugiperda* females in the Western Hemisphere contain similar sex pheromone compounds as in Republic of Benin (Haenniger et al. 2020), the response and capture of *S. frugiperda* males using commercial lures with some of these compounds have been erratic (Meagher et al. 2019; Tepa-Yotto et al. 2022). Thus, effective, local, regional and continental species-specific detection, monitoring and pest management should be developed as solid bases of IPM strategies (Saveer et al. 2023).

The sex pheromone of *S. frugiperda* in the Americas consists of (Z)-9-tetradecenyl acetate (Z9-14:OAc) and (Z)-7-dodecenyl acetate (Z7-12:OA) which have been found to be important for the attraction of conspecific males (Tumlinson et al. 1986; Andrade et al. 2000; Unbehend et al. 2013, 2014; Jiang et al. 2022). Adding (Z)-11-hexadecenyl acetate (Z11-16:OAc) increased male attraction in Pennsylvania (Fleischer et al. 2015), and also elicited a small electroantennogram response to *S. frugiperda* males in Yunnan Province (Jiang et al. 2022). In Brazil, (E)-7-dodecenyl acetate (E7-12:OAc) was identified as an additional active sex pheromone component (Batista-Pereira et al. 2006; Cruz-Esteban et al. 2020). In Africa, *S. frugiperda* monitoring studies with pheromone lures have revealed varying results (Meagher et al. 2019; Koffi et al. 2021; Tepa-Yotto et al. 2022). For example, in Benin, when Tepa-Yotto et al. (2022) compared the attraction of *S. frugiperda* males among three blends, i.e., a four-component blend containing Z9-14:OAc, Z7-12:OAc, Z11-16:OAc and (Z)-9-dodecenyl acetate (Z9-12:OAc), a three-component blend (without Z9-12:OAc) and a two-component blend containing only Z9-14:OAc and Z7-12:OAc, they found that the 4-component blend attracted the highest number of *S. frugiperda* males, irrespective of the conditions of the experiment, while the 2-component blend was the most selective, as the percentage of bycatches was the lowest (Tepa-Yotto et al. 2022). Conversely, Koffi et al. (2021) found that the 3-component lure containing Z9-14:OAc, Z7-12:OAc, Z11-16:OAc was more attractive than the 4-component lure (adding Z9-12:OAc) in the neighboring country Togo. Thus, within African, male *S. frugiperda* responses to female sex pheromones may differ.

To assess whether males from different populations show different physiological responses to the identified sex pheromone compounds, we investigated the antennal responses of males from three African populations and an American population to all identified synthetic sex pheromone compounds. We also determined whether male responses varied depending on strain variation in *S. frugiperda*. Since we genotyped the males with the COI marker, which is mitochondrial (Nagoshi et al. 2022), we use the term ‘mitotype’ in the remainder of this manuscript. Specifically, we evaluated variability in the antennal response of *S. frugiperda* males that were collected as larvae from Florida, Benin, Nigeria and Kenya and from (C)- and (R)- mitotyped males from Benin and Florida, to E7-12:OAc, Z7-12:OAc, Z9-12:OAc, Z9-14:OAc and Z11-16:OAc.

## Methods and Materials

### Insect Collection and Rearing

The *S. frugiperda* populations from Benin originated from larvae, collected from maize fields and alternative host plants in South and Central Benin by the International Institute of Tropical Agriculture (IITA), Benin in July 2020, from which a laboratory population was established at the University of Amsterdam. This population was mixed with field specimens from Azowlisse, Benin, which were collected in December 2020. In July 2021 and October 2022, new specimens from the lab population from IITA were mixed into the lab population at the University of Amsterdam. The Kenyan population was collected from the lab population of International Centre of Insect Physiology and Ecology (*icipe*), Kenya. Specimens were collected from several areas in Kenya at different time points. At icipe, the population was kept in temperature of 25 ± 2 °C, 70% ± 5% relative humidity and a 12:12 h (light: darkness) photoperiod and on an artificial diet. Samples of this lab population were received at the University of Amsterdam in December 2020 and August 2022.The Nigerian population was collected from maize fields in Southern Oyo State and from IITA, Ibadan, Nigeria, between January 2022 and May 2022. To compare responses of African *S. frugiperda* males to American *S. frugiperda* males, Florida (C)- and (R)- strains were also reared at the University of Amsterdam, with populations obtained from the Max Planck Institute of Chemical Ecology (MPICE), Jena, Germany. These populations were collected as larvae in September 2018 near Citra (Florida, USA) in a corn field and in January 2019 in pasture (rice-strain) near Ona (Florida, USA) and bred in MPICE in climate chambers at 26 °C, 55% relative humidity and light:darkness (L:D) 14:10.

The immature stages (eggs and larvae) of all the populations were further reared on an artificial pinto bean diet in climate chambers at 25 °C and humidity of 60–65%, with reversed light/dark cycle and 14:10 light/dark photoperiod at the laboratory at the University of Amsterdam (IBED). The adults were fed with 10% sugar water, ad libitum. Male and female insects were used for mitotype identification, while 2–5 day-old virgin males were used for gas chromatography-electroantennogram detector (GC-EAD) experiments.

### Mitochondrial Identification of African Population

Since the invasive populations of *S. frugiperda* are mixed in their nuclear genome, but do consist of two mitotypes (Yainna et al. 2021; Tay et al. 2022), we determined whether males with different mitotypes show different electrophysiological responses. Mitotype identification was done by screening adults for the mitochondrial marker (COI) that is diagnostic for both strains in North and South America (Nagoshi et al. 2006a, b; Nagoshi 2010) and generally used in other populations as well (Tay et al. 2022). DNA extractions were performed in a 96-well plate using Chelex 100 Resin (Bio-Rad Laboratories, Hercules, CA, USA). One adult leg was put in one well together with two metal beads and 300 µl 10% Chelex (diluted in ddH_2_O). The tissue was homogenized in a tissue lyser for 4 min at 30 Hz. Samples were heated for 30 min at 95 °C and 300 rpm spinning, after which they were frozen at − 20 °C overnight. Each plate was thawed, mixed, and centrifuged at 4000 rpm for 30 min. The supernatant was filtered through a fritted deep well filter plate (Thermo Fisher Scientific, Waltham, MA, USA) and used for mitotype analysis. Identification of the mitochondrial COI gene was performed as described by Unbehend et al. (2013) and summarized here. After amplification of the COI gene, two strain-specific digests with MspI and SacI (New England Biolabs, Ipswich, MA, USA) were conducted to analyze the strain-affiliation via gel electrophoresis.

To compare EAG responses between the mitotypes, we used mitotyped males from Florida and Benin. The sampled populations from Benin contained both (C)- and (R)- mitotypes, while sampled populations from Nigeria were all (R)- mitotype, and Kenyan populations were all (C)-mitotype (see supplementary Photo S1-S4 and Supplementary Table S1). In our preliminary analysis, we observed no significant differences in the responses between Benin (R)—and Nigerian (R)—mitotyped males.

### Preparation of the Multicomponent Blends

All used synthetic sex pheromone compounds of *S. frugiperda*were purchased from Pherobank (WijkbijDuurstede, The Netherlands), i.e., E7-12:OAc, Z7-12:OAc, Z9-12:OAc, Z9-14:OAc and Z11-16:OAc with > 98% purity. To determine the antennal responses to these compounds, two multicomponent blends (MCBs) were prepared, one with E7-12:OAc and one with Z7-12:OAc in combination with Z9-12:OAc, Z9-14:OAc, Z11-16:OAc (Supplementary Table S2). This allowed us to conveniently distinguish antennal responses between E and Z7-12:OAc.

A stock solution of 10 µg/µl in hexane was made for each synthetic pheromone compounds from which the two MCBs (i.e.one including E7-12:OAc and the other including Z7-12:OAc) were made by diluting 1 µl each of the four synthetic pheromone compounds into 500 µl of hexane. Each of concentrations of 1 ng/µl, 3 ng/µl, 10 ng/µl and 20 ng/µl was obtained in a serial dilution and samples were put in the vials and kept in -20˚C until the time of chemical analysis.

### GC-EAD Measurements

To measure the electrophysiological responses of male antennae, the two MCBs in the four different concentrations were used randomly on 2 to 4-day-old virgin males from Benin, Kenya, Nigeria and Florida (C-type and R-type). Live male insect were individually placed in a plastic pipette tip and one antenna was immobilized with a small strip of parafilm pressing the antennal base against the head. Electrical contact was made using silver wires inserted in glass microelectrodes (GC150TF-10; Warner Instruments, Hamden, CT, USA) with insect Ringer’s solution. The recording electrode was inserted at the base of the antenna and the reference electrode made contact with the cut antennal tip. The amplitude of the EAG was measured using an IDAC-4 amplifier equipped with a high impedance (> 10^9^ Ohm) head stage and recorded with GC-EAD/2014 software (Syntech, Kirchzarten, Germany). An Agilent 7890B gas chromatograph (Agilent, Wilmington, DE, USA) equipped with an Agilent Cool On-Column inlet, was coupled to the EAG setup to deliver odor stimuli. Details of the GC-EAD set up are in the supplementary S2.

To check the longevity of the whole insect preparation, a reference stimulus (containing Z3-6:OH, 10^–3^ v/v (4.2 µg) in paraffin oil) was delivered for 0.5 s from a filter paper strip in a Pasteur pipette by a CS-55 stimulus controller (Syntech). The preparations proved to be stable, and could have been used easily for a full day. Among sampled references, there was no significant difference between EAG responses before and after the each run (*t*-test, *n* = 14, *P* > 0.05, *df* = 1).

### Statistics and Data Analyses

To analyze the antennal responses to the pheromone compounds, we built linear mixed effect models (Lindstrom and Bates 1988) using the *lme* function (Pinheiro et al 2022) implemented in R (R Core Team 2022). Mixed models, with individual insect as a random factor and compound, concentration, population and (when available) mitotype as fixed factors, allowed us to separate the (random) variation between individual insect from the variation caused by the (fixed) factors of interest.

We built three separate models (Supplementary Table S3). The first model contained compound, concentration and population as predictors, with 498 EAD responses from 25 male insects. In addition to male insects from Florida, Kenya and Nigeria, this set comprises only insects with unknown mitotype from Benin. This is because the mitotyped insects were not run in the same experiment set and therefore may not be strictly comparable to the other data. A second model contained mitotype as explanatory variable. The model was fitted with 397 cases from 19 males for which mitotype information was available, that is, Benin and Florida. Finally, since the results indicated that there was no significant difference between the African populations, a third model was constructed to compare EAD responses on a continental scale (Africa vs America). This dataset contained in total 697 EAD responses from 34 males, this set contained all available data.

For each model, we started with a full model that contained the random factor, all the fixed factors and their interactions. Model selection of better fitting model was done based on Akaike’s Information criterium (AIC) (Sakamoto et al. 1986). All models were fit by maximum likelihood to allow model comparison using AIC. Post hoc comparisons of mean values from the final models was done with Least-squares means (Searle et al. 1980) as implemented in the R package *emmeans* (Lenth 2023) with Tukey multiplicity adjustments.

## Results

### Electrophysiological Responses to Synthetic Sex Pheromone Compounds

**Fig. 1:**
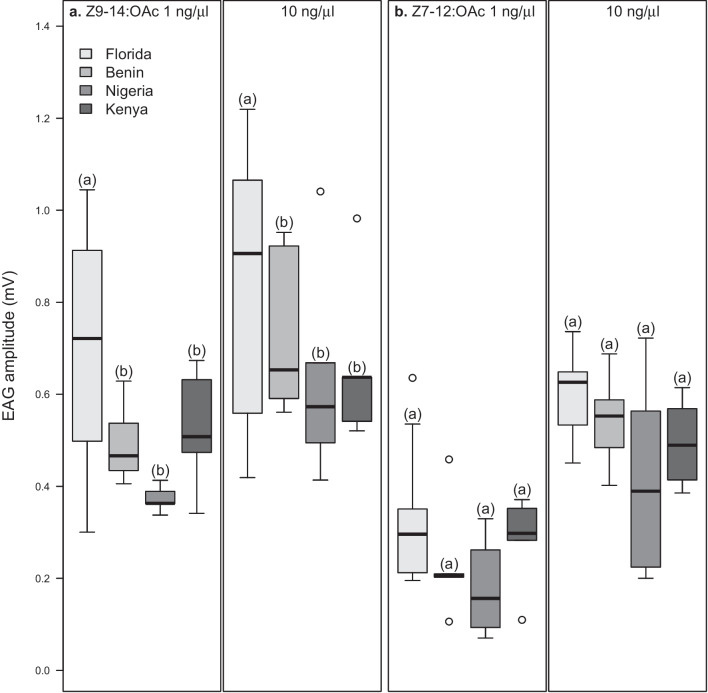

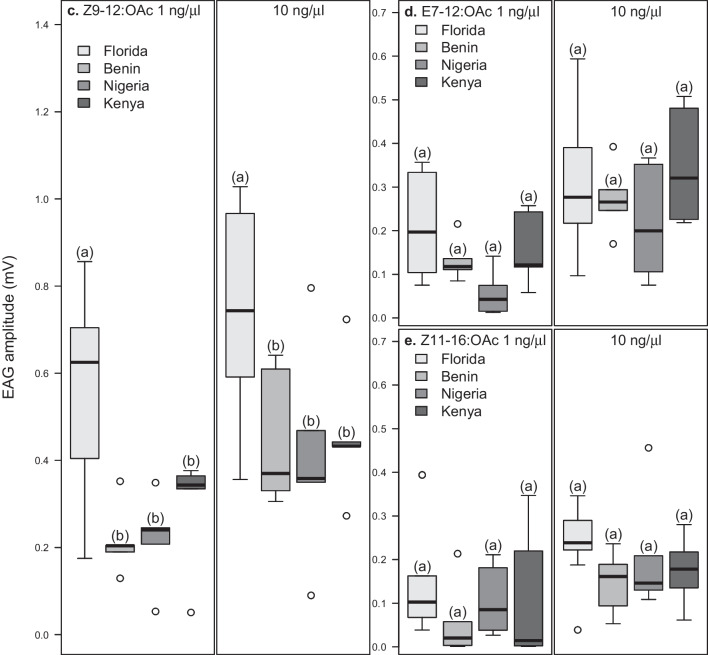
Responses of *Spodoptera frugiperda* males to different doses of sex pheromone compounds**.** Boxes represent the lower and upper quartiles of responses to (**a**) Z9-14:OAc (**b**) Z7-12:OAc (**c**) Z9-12:OAc (**d**) E7-12:OAc, and (**e**) Z11-16:OAc. Whiskers on boxes indicate the minimum and maximum values, excluding outliers. Middle line represents median of values (*n* = 10 in Florida, except in Z7-12:OAc and Z9-12:OAc at 1 ng/µl (where *n* = 9); *n* = 5 in Benin, Nigeria and Kenya); significant differences within regions are indicated by different letters, *P* < 0.05

When checking the electrophysiological responses of *S. frugiperda* males from the different regions, we found significant effects of geographic regions, the compounds, concentrations, as well as interactions between geographic region and compounds and between concentration and compound (Fig. 1). The major compound, Z9-14:OAc, and Z9-12:OAc elicited significant responses among the different regions (Fig. 1a, c), as Florida male responses were higher than male responses from Benin, Nigeria and Kenya, due to a significant interaction between population and compound (*P* < 0.05, *df* = 442). However, Benin, Nigerian and Kenyan males responded equally to Z9-14:OAc and Z9-12:OAc (*P* > 0.05, *df* = 442). In all regions, *S. frugiperda* males responded similarly (*P* > 0.05, *df* = 442) to Z7-12:OAc, E7-12:OAc and Z11-16:OAc (Fig. 1b, d, e).

**Fig. 2:**
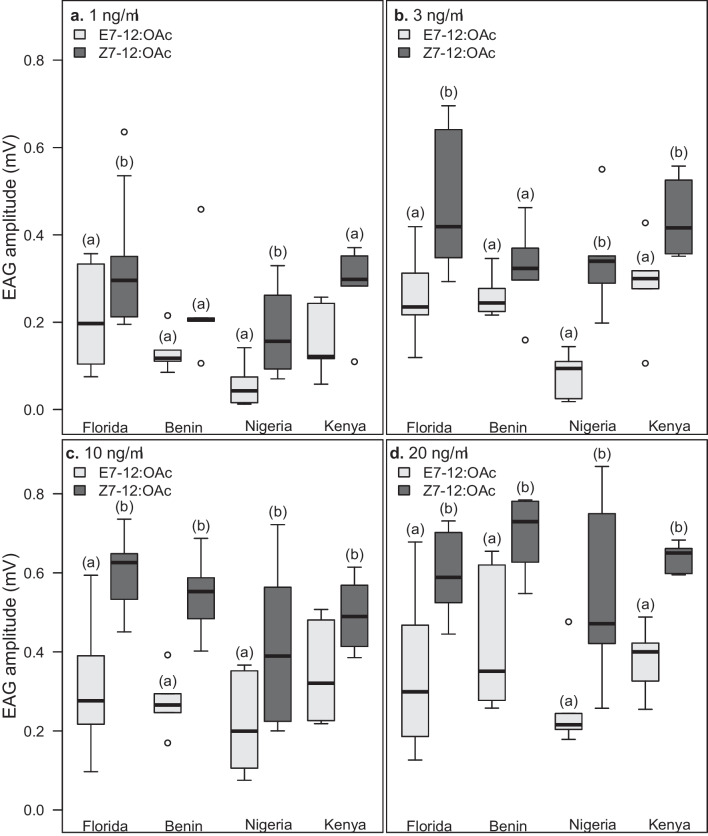
Responses of *Spodoptera frugiperda* males to Z7-12:OAc and E7-12:OAc at different doses. Boxes represent the lower and upper quartiles of responses to Z7-12:OAc and E7-12:OAc. Whiskers on boxes show the minimum and maximum values, excluding outliers (*n* = 10 in Florida, except in Z7-12:OAc at 1 ng/µl (where *n* = 9); *n* = 5 in Benin, Nigeria and Kenya); significant differences within regions are indicated by different letters, *P* < 0.05

In assessing whether males responded differently to the two synthetic isomers, Z7-12:OAc and E7-12:OAc, we plotted the responses to both compounds from each region and at different concentrations in separate boxplots (Fig. 2). Overall, Z7-12:OAc gave higher antennal responses than E7-12:OAc in all the regions (*P* < 0.05, *df* = 632, Fig. 2a–d). Also, only Florida and Nigerian males responded significantly to the two compounds at 1 ng/µl, while Kenyan males responded more to Z7-12:OAc than E7-12:OAc at 3 ng/µl. At higher concentrations (10 ng/µl and 20 ng/µl), males from all the regions (Florida, Benin, Nigeria and Kenya) showed significantly higher antennal responses to Z7-12:OAc than E7-12:OAc (*P* < 0.05, *df* = 632).

**Fig. 3:**
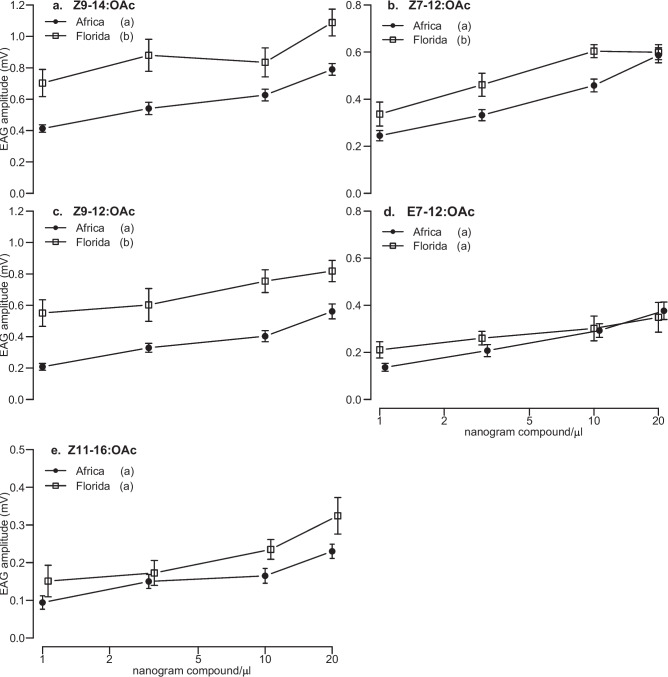
The electroantennography (EAG) dose-response curves of the antennae of *Spodoptera frugiperda* males from combined Africa. Male responses to (**a**) Z9-14:OAc (**b**) Z7-12:OAc (**c**) Z9-12:OAc (**d**) E7-12:OAc and (**e**) Z11-16:OAc. Error bars indicate mean (±SEM) of EAG amplitudes (mV) (*n* = 25 in Africa, except in Z7-12:OAc at 1 ng/µl (where *n* = 24); *n* = 10 in Florida (except in Z7-12:OAc and Z9-12:OAc at 1 ng/µl, where *n* = 9); significant differences within continents are indicated by different letters, *P* < 0.05

Since there was no difference in male responses to the compounds in the three African populations (Fig. 1), we combined these responses to a so-called ‘African’ response and compared their overall additive responses to the Florida (American) responses. Our results revealed some differences across the two continents (Fig. 3a–e). Specifically, EAG responses evoked by the major compound, Z9–14:OAc, the critical secondary compound, Z7–12:OAc and Z9–12:OAc were significantly higher in American males than in African (Benin, Kenyan and Nigerian) males (*P* < 0.05, *df* = 640). However, we found no differences in responses to E7-12:OAc and Z11-16:OAc between the continents.

**Fig. 4:**
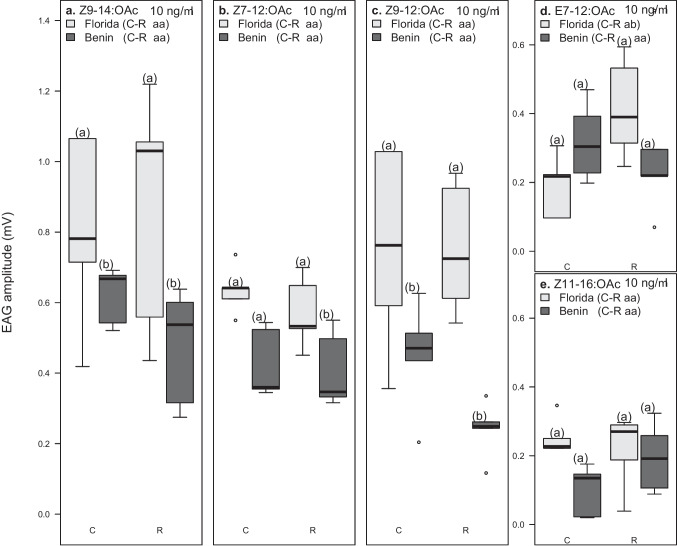
Responses of *Spodoptera frugiperda* Florida and Benin C- and R-type males to different doses of sex pheromone compounds. Boxes represent the lower and upper quartiles of responses to (**a**) Z9-14:OAc (**b**) Z7-12:OAc (**c**) Z9-12:OAc (**d**) E7-12:OAc and (**e**) Z11-16:OAc. Whiskers on boxes indicate the minimum and maximum values, excluding outliers (*n* = 5); significant differences within groups are indicated by different letters, *P* < 0.05

When analyzing variation in mitotyped male responses, we found a significant effect of compound, geographic region and a three-way interaction between compound, mitotype, and geographic region (*P* < 0.05, *n* = 5, *df* = 358; Fig. 4). Specifically, Florida (C)-type males showed significantly lower response to E7-12:OAc than Florida (R)-type males (*P* < 0.05, *n* = 5,*df* = 358), while both mitotypes responded similarly to other compounds. Each of the five synthetic compounds also evoked similar responses in Benin (C)-type and (R)-type males. Between Benin (R)-type and Florida (R)-type males, we observed significant higher responses of Florida (R)-type males to Z9-14:OAc (*P* < 0.05, *n* = 5, *df* = 358), Z7-12:OAc (*P* < 0.05, *n* = 5, *df* = 358)and Z9-12:OAc (*P* < 0.05, *n* = 5, *df* = 358) (Fig. 4a–c). Also, Florida (C)-type males showed significantly higher responses than Benin (C)-type males to Z9-14:OAc and Z9-12:OAc (*P* < 0.05, *n* = 5, *df* = 358; Fig. 4a, c).

## Discussion

In this study, we investigated the variation in electrophysiological responses of *S. frugiperda* males from Florida, Benin, Nigeria and Kenya to sex pheromone compounds, and found geographic variation in responses of males to Z9-14:OAc and Z9-12:OAc in all regions. Z7-12:OAc elicited males responses that were more than those of E7-12:OAc in the regions.

We also found geographical and mitotype differences in responses to some sex pheromones compounds, whereby (R)-mitotype males showed higher responses to E7-12:OAc than their conspecific (C)-mitotype from Florida and higher responses to Z7-12:OAc, Z9-14:OAc and Z9-12:OAc than (R)-mitotype males from Benin.

### Geographic Variation in Male Responses

Clearly, *S. frugiperda* males from the different regions responded to all synthetic sex pheromone compounds, even though to a varying degree. Our finding that Florida males showed higher responses to the major compound, Z9-14:OAc than males from Benin, Nigerian and Kenyan, corresponds to previous results by Haenniger et al. (2020), where Florida males also elicited greater EAG responses to Z9-14:OAc than Benin and Nigerian males. Since it is widely agreed that Z9-14:OAc is the major sex pheromone component needed to effectively attract *S. frugiperda* males (Tumlinson et al. 1986; Andrade et al. 2000; Meagher and Nagoshi 2013; Meagher et al. 2019; Haenniger et al. 2020), it is interesting that African male responses are generally lower than those from Florida. The lower EAG responses of African male populations compared to Florida populations may be due to the low genetic variation observed in Africa population (Nagoshi et al. 2022), suggesting a single introduction of a small invasive population through western Africa, which is supported by the findingthat all haplotypes were similar and consistent with a common source population (Nagoshi et al. 2019a, b; Nagoshi et al. 2018; Nagoshi et al. 2022).

Similarly, Z9-12:OAc elicited greater EAGs in Florida males than in Benin, Nigerian and Kenyan males. The activity of Z9-12:OAc as a pheromone component component has has only occasionally been documented, although Tepa-Yotto et al. (2022) found that the addition of Z9-12:OAc to a four-component pheromone blend improved male attraction. Possibly, adding Z9-12:OAc to the sex pheromone blend will increase male attraction in Africa.

A generally low EAG amplitude was observed in response to Z11-16:OAc in all the regions tested, which is consistent with previous findings (Malo et al. 2004; Unbehend et al. 2013; Jiang et al. 2022). Z11-16:OAc does not seem to be a sex pheromone component in the American population (Unbehend et al. 2014) and therefore may not increase male attraction in the African population either (Tepa-Yotto et al. 2022). Moreover, the addition of Z11-16:OAc to lures caused large numbers of bycatches of non-target moths, particularly *Mythimna loreyi* (Duponchel) in West Africa (Meagher et al. 2019; Tabata et al. 2022), indicating that *S. frugiperda* lures in Africa should be developed without Z11-16:OAc.

### Variation in Response to E7-12:OAc and Z7-12:OAc

We found interesting variations in male responses to the two isomeric compounds, Z7-12:OAc and E7-12:OAc across the concentrations tested. Overall, all *S. frugiperda* males showed higher EAG responses to Z7-12:OAc than to its isomer, E7-12:OAc. So far, E7-12:OAc has been reported only within the female glands of Brazilian *S. frugiperda* populations (Batista-Pereira et al. 2006) and more interestingly, was behaviorally active on males from that region (Batista-Pereira et al. 2006; Cruz-Esteban et al. 2018). Whether E7-12:OAc is absent in female glands in African or other American regions is mostly still unclear, as is the male response in other regions. Unbehend et al. (2014) did find that (C)-type males from Peru are only attracted to a blend containing Z7-12:OAc, but not to a blend with E7-12:OAc, while males in North Carolina did not differentiate between the two isomers. As chromatographic separation of the two isomers is difficult, it is possible that this compound has remained undetected in other studies. However, the lower EAG responses of the males to E7-12:OAc than Z7-12:OAc from all four regions suggests that the latter may be more important than the former in male attraction in all regions.

### Variation in Response Between Africa and Florida

Our finding that *S. frugiperda* males from Benin, Kenya and Nigeria showed similar EAD responses to all pheromone compounds, is comparable to the reports of Haenniger et al. (2020) where Benin and Nigerian *S. frugiperda* males exhibited similar EAG amplitudes to the five known sex pheromone synthetic compounds. These results suggest that *S. frugiperda* males may not show geographic variation within the Africa continent. However, geographic variation between continents seems to occur, as we found intercontinental differences in the male responses towards Z9-14:OAc, Z7-12:OAc and Z9-12:OAc, probably due to the lower genetic variability among the African *S. frugiperda* than those of Florida.

### Variation in Inter-type and Intra-type Geographic Males

Interestingly, when comparing the mitotypes, we found that Florida (C)- and (R)-type males differed significantly in their response to E7-12:OAc and not any other pheromone compounds. The fact that there was no type-specific differential response to all but one pheromone compound in Florida males, and no type-specific differential response to any pheromone compound in Benin males suggests that males of both mitotypes have similar response range and are not differentiated with respect to antennal response at the doses tested. Field trapping experiments with Florida population also showed that both strains were similarly attracted to pheromone lures (Unbehend et al. 2013, 2014; Kenis et al. 2023). Also in Kenya, both corn and rice mitotypes were equally attracted to the different commercial pheromone lures when tested in replicated field trials (Sisay et al. 2024).

In conclusion, we found that electrophysiological responses of *Spodoptera frugiperda* males to sex pheromone compounds differ between Florida and Africa population, but responses do not differ within the African continent. These results suggests that pheromone lures may have to be adjusted for monitoring of *Spodoptera frugiperda* in Africa compared to America, but do not have to be adjusted for specific regions within the African continent. Our results also indicate that within the Africa region, mitotype differences do not seem to occur, at least in terms of physiological responses, which makes it less likely that one of the mitotypes will not be detected. As current commercial lures are not working optimally (Meagher et al. 2019; Tepa-Yotto et al. 2022), we do recommend to specify lure compositions towards blends that mimick the female sex pheromone composition of *S. frugiperda* in Africa.

## Supplementary Information

Below is the link to the electronic supplementary material.

Supplementary file1 (DOCX 2679 KB)

## Data Availability

Data supporting the findings of this study will be made available on request.
