# Supplementary material for: Region-Specific Variation in the Electrophysiological Responses of Spodoptera frugiperda (Lepidoptera: Noctuidae) to Synthetic Sex Pheromone Compounds
Source: J Chem Ecol. 2024 Feb 29;50(11):631–42. doi: 10.1007/s10886-024-01479-w (PMC11543750; doi:10.1007/s10886-024-01479-w)
Supplement: Supplementary file 1 — Supplementary file1 (DOCX 2679 KB) [file 10886_2024_1479_MOESM1_ESM.docx]

**Supplementary Material**

**Region-specific variation in the electrophysiological responses of *Spodoptera frugiperda* (Lepidoptera: Noctuidae) to synthetic sex pheromone compounds**

*Mobolade D. Akinbuluma^1,2^, Renée A.H. van Schaijk^1^, Peter Roessingh^1^, Astrid T. Groot^1^

*1. University of Amsterdam, Department of Evolutionary and Population Biology, The Netherlands*

*2. University of Ibadan, Department of Crop Protection and Environmental Biology, Ibadan, Nigeria*

Corresponding author. Tel.:+31 6 86 279835, e-mail: [m.d.akinbuluma@uva.nl](mailto:m.d.akinbuluma@uva.nl)

**Supplementary Photo S1-S4.** Gel images of mitotyped populations of sampled *Spodoptera frugiperda* from Benin (A), Kenya (B, C) and Nigeria (D). The PCR product was cut with two restriction enzymes, MspI + SacI (New England Biolabs, Ipswich, MA, USA). The size markers A 100 base pair ladder was used and the indicated fragment sizes are the sizes from Nagoshi et al. (2006). For the (C)- mitotype the original fragment (568 bp) and the large cut fragement (462 bp) are expected, for the (R)- mitotype, the PCR product (568 bp) and two restriction fragments (413 and 155 bp).

**Supplementary Table S1**

Summary of identified mitotypes by population. Failed amplifications or restrictions are indicated with X

| **Population** | **Mitotypes** | | | **Percentage** | |
| --- | --- | --- | --- | --- | --- |
|  | **C** | **R** | **X** | **% C** | **%R** |
| Benin | 9 | 4 | 0 | 69.2 | 20.8 |
| Kenya | 57 | 0 | 26 | 100 | 0 |
| Nigeria | 0 | 21 | 9 | 0 | 100 |

**Supplementary Table S2 Chemical components of Multicomponent blends (MCBs) of**

***S. frugiperda* sex pheromone compounds**

| **Sex pheromone**  **Compounds** | **Molecular**  **Formula** | **MCB 1** | **MCB 2** |
| --- | --- | --- | --- |
| E7- 12:OAc | C_14_H_26_O_2_ | x ng/µl | - |
| Z7-12:OAc | C_14_H_26_O_2_ | - | x ng/µl |
| Z9-12:OAc | C_14_H_26_O_2_ | x ng/µl | x ng/µl |
| Z9-14:OAc | C_16_H_30_O_2_ | x ng/µl | x ng/µl |
| Z11-16:OAc | C_18_H_34_O_2_ | x ng/µl | x ng/µl |

**Supplementary S2 Details of GC-EAD**

The Cool on Column inlet was connected to a 2-m-long, 0.53-mm retention gap (Phenomenex, Utrecht, the Netherlands) and connected with a glass press-fit (Techrom, Purmerend, the Netherlands) to a 30-m EC-5 column, 0.25 mm inner diameter,0.25*µ*m film (Fisher Scientific Pittsburgh, PA, USA). The effluent from this column was split using a Dean’s switch, controlled with N_2,_ while the pressure on both control inputs was kept equal to create a 1:1 split ratio. The two outlets of the switch were connected to sections of 100 cm × 0.25-mm-diameter deactivated capillary column (Phenomenex), one going to the standard flame ionization detector (FID) set at 250 °C, the other leaving the GC oven via a 30-cm-long heated transfer-line (Syntech) set to 240 °C.The capillary emerging from the transfer-line protruded through a 0.5 mm hole into a 1-cm-wide, L-shaped glass tube (Syntech) carrying a charcoal filtered and humidified airstream of 1 l/min from a CS-55 airflow controller (Syntech) to the preparation that was placed directly in front of the outlet. The oven temperature program started at 60°C for 10 seconds and stepped to 80°C. After 5 minutes, temperature was programmed to increase to a maximum of 250°C at 10°C/min.

**Supplementary Table S3 Overview of the fixed factors in the best fitting models selected by the Akaike's Information criterium (AIC). In all models, the response was EAD amplitudes and insect ID was incorporated as random factor.**

| Model | Model (fixed factors) | N (cases) | N (males) | AIC |
| --- | --- | --- | --- | --- |
| Regional  Comparison | concentration, compound, population, compound:population compound:concentration | 498 | 25 | -406.5798 |
| Mitotype  Comparison | concentration, compound, population, compound:strain:population | 397 | 19 | -363.0666 |
| Continent comparison | concentration, compound, continent, compound:continent, compound:concentration | 697 | 34 | -654.1676 |
